# Characterization of Pellicle Inhibition in *Gluconacetobacter xylinus* 53582 by a Small Molecule, Pellicin, Identified by a Chemical Genetics Screen

**DOI:** 10.1371/journal.pone.0028015

**Published:** 2011-12-09

**Authors:** Janice L. Strap, Andrew Latos, Isaac Shim, Dario T. Bonetta

**Affiliations:** Faculty of Science, University of Ontario Institute of Technology, Oshawa, Ontario, Canada; Aarhus University, Denmark

## Abstract

Pellicin ([2E]-3-phenyl-1-[2,3,4,5-tetrahydro-1,6-benzodioxocin-8-yl]prop-2-en-1-one) was identified in a chemical genetics screen of 10,000 small molecules for its ability to completely abolish pellicle production in *Gluconacetobacter xylinus*. Cells grown in the presence of pellicin grew 1.5 times faster than untreated cells. Interestingly, growth in pellicin also caused *G. xylinus* cells to elongate. Measurement of cellulose synthesis *in vitro* showed that cellulose synthase activity was not directly inhibited by pellicin. Rather, when cellulose synthase activity was measured in cells that were pre-treated with the compound, the rate of cellulose synthesis increased eight-fold over that observed for untreated cells. This phenomenon was also apparent in the rapid production of cellulose when cells grown in the presence of pellicin were washed and transferred to media lacking the inhibitor. The rate at which cellulose was produced could not be accounted for by growth of the organism. Pellicin was not detected when intracellular contents were analyzed. Furthermore, it was found that pellicin exerts its effect extracellularly by interfering with the crystallization of pre-cellulosic tactoidal aggregates. This interference of the crystallization process resulted in enhanced production of cellulose II as evidenced by the ratio of acid insoluble to acid soluble product in *in vitro* assays and confirmed *in vivo* by scanning electron microscopy and powder X-ray diffraction. The relative crystallinity index, RCI, of pellicle produced by untreated *G. xylinus* cultures was 70% while pellicin-grown cultures had RCI of 38%. Mercerized pellicle of untreated cells had RCI of 42%, which further confirms the mechanism of action of pellicin as an inhibitor of the cellulose I crystallization process. Pellicin is a useful tool for the study of cellulose biosynthesis in *G. xylinus*.

## Introduction

Cellulose is an abundant and natural polymer of interest not only because of its role in the carbon cycle [1] but also due to its many industrial uses such as energy [2], paper [3] and materials [4]. In addition, cellulose is a crucial component of the extracellular matrix of many commensal and pathogenic biofilm-forming bacteria underscoring the importance of studying bacterial cellulose synthesis. *Gluconacetobacter xylinus* (formerly *Acetobacter xylinum*) is a Gram-negative, aerobic bacterium that serves as a model organism for the study of bacterial cellulose biosynthesis [5]. *G. xylinus* abundantly produces an extracellular and pure form of crystalline cellulose as a pellicle at the air-liquid interface of statically grown liquid cultures. It has been hypothesized that pellicle cellulose plays a storage role and can be utilized under starvation conditions [6]. Pellicle utilization would require exo- and endo-glucanases, both of which are detectable in culture broth [7]. The pellicle can provide protection to unfavorable environmental changes such as a decrease in water content, variation in pH, toxic substances and UV radiation [8]. Furthermore, pellicle production enhances colonization of plants, providing protection from competitors on the same substrate [8].

The two major allomorphs of cellulose, designated cellulose I and cellulose II are distinguishable by X-ray diffraction, NMR [9], Raman spectroscopy and infrared analysis [10]. Cellulose I, a highly crystalline form produced by plants is also the form synthesized by *G. xylinus* in static culture in which the linear β-1,4-glucan chains are oriented parallel to one another with the same polarity whereas the β-1,4-glucan chains of cellulose II are arranged randomly making the cellulose structure amorphous [11] and more easily degradable. Cellulose I can be converted to cellulose II by mercerization (treatment with 18% NaOH) [12].

While many of the key genes involved in cellulose biosynthesis and regulation have been identified, the biochemical details of biosynthesis are not clear. Genes known to be involved in bacterial cellulose synthesis encode an operon composed of *bcsA*, *bcsB*, *bcsC*, and *bcsD* [13], [14], [15]. Recently it has been shown that there are two additional copies of each gene of the operon with the exception of *bcsD* [16]. BcsA catalyzes the polymerization of single monomer units of uridine diphosphoglucose (UDP-Glc) into glucan chains while BcsB likely regulates activity of cellulose synthesis by binding 3′,5′-cyclic diguanylic acid (c-di-GMP) [17], [18], an allosteric regulator, via a PilZ domain [19]. The synthesis and degradation of c-di-GMP are carried out by a diguanylate synthase (Dgc) and a phosphodiesterase (Pde), respectively [20]. Notably, the genes encoding these enzymes are located on three distinct yet highly homologous *cdg* operons [21]. The protein products of these genes contain amino acid sequence motifs referred to as GGDEF and EAL domains [22]. Proteins containing these sequence motifs mediate signal transduction [23] underscoring the importance of extracellular matrix production in response to environmental signals. While it is clear that c-di-GMP exerts a regulatory influence, the mechanism by which it does remains unsolved. For example, the disruption of *dcg1* unexpectedly results in a mutant that produces comparable amounts of cellulose compared to wildtype under static conditions but more cellulose when grown in agitated culture [24]. Contradictory results were obtained by Tal *et al.* [20] who reported that disruption of *dcg1* decreases cellulose production.

The proposed organization of polypeptides in the cellulose-synthesizing complex of *G. xylinus* consists of the catalytic domains being located in the cytoplasmic membrane along with the c-di-GMP activator binding polypeptides. The BscC polypeptide has been proposed to form the major channel for export of the cellulose from the cytoplasmic membrane to the cell surface. The BcsD polypeptide, for which the crystal structure [25] has recently been elucidated, is thought to bind to the other two gene products either within the channel or on the outer surface of the membrane thereby influencing crystallinity; although this has not been shown experimentally. Two other protein products encoded by genes upstream of the operon are implicated in the cellulose synthesis pathway: CMCax, an endoglucanase that has cellulose-hydrolyzing activity [26], [27], [28] and CCPax, believed to be involved in the crystallization process [29]. It has yet to be determined whether BcsD and CCPax interact to influence the crystallization of cellulose and importantly, if either directly participate in the process. How cellulose is crystallized at the cell surface remains to be determined.

In the present study, we used a chemical genetics approach [30] to identify a small molecule inhibitor of bacterial cellulose biosynthesis by conducting a high throughput perturbation study of cellulose biosynthesis using a library of small organic molecules. We took advantage of the abundant extracellular pellicle formation of *G. xylinus* to design the high throughput assay for cellulose inhibition. One molecule was identified by screening a collection of 10,000 molecules for its ability to inhibit pellicle formation. From this screen, we identified a potent inhibitor that we named pellicin. We report here the analysis of pellicin action which acts late in the cellulose biosynthetic pathway by affecting cellulose crystallinity. To our knowledge, this is the first use of chemical genetics to investigate the cellulose biosynthesis pathway in *Gluconacetobacter xylinus*.

## Results

### Identification of cellulose inhibitor pellicin by chemical genetics screen

**Figure 1:**
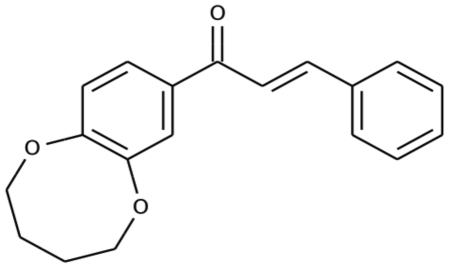
Chemical structure of pellicin.

**Figure 2:**
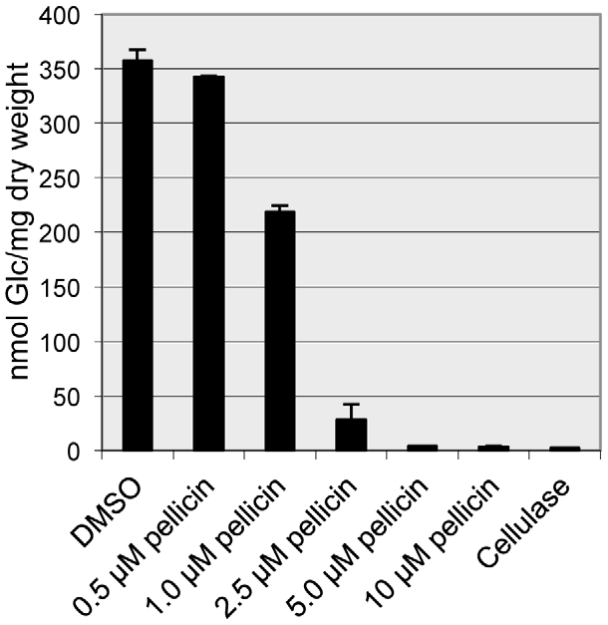
Dose-response curve for pellicin inhibition of *G. xylinus* pellicle. In each case *G. xylinus* cultures were grown from a starting culture with an OD_600_ = 0.15 and either DMSO or pellicin was added to the culture and allowed to grow without shaking for 2 days at 30°C. Cellulase was added to 0.1% (w/v). Values are averages ± SD; n = 3.

In an effort to generate innovative tools for the study of cellulose synthesis in *G. xylinus* ATCC 53582, a chemical genetics approach was used to identify inhibitors of pellicle formation. Ten thousand chemically diverse small molecules were tested in a high throughput screen. *G. xylinus* exhibited a range of phenotypes when grown in the presence of these small molecules from no inhibition of pellicle production, to partial pellicle inhibition to complete inhibition of pellicle formation. One inhibitor identified in the screen, [2E]-3-phenyl-1-[2,3,4,5-tetrahydro-1,6-benzodioxocin-8-yl]prop-2-en-1-one (Fig. 1) was able to completely abolish pellicle production at low concentrations. Pellicle inhibition was examined for a range of concentrations using the new inhibitor, which indicate that 5 µM of pellicin abolishes pellicle production (Fig. 2). We have named this compound ‘pellicin’ to describe its pellicle inhibiting activity.

### Pellicin abolishes pellicle production but does not inhibit cell growth

**Figure 3:**
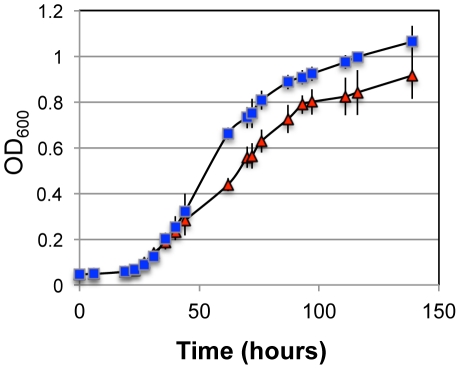
The influence of pellicin on *G. xylinus* growth. Pellicin does not affect the viability of *G. xylinus*. *G. xylinus* was grown at 30°C in Schramm-Hestrin broth containing 0.1% cellulase and either 10 µM pellicin (▪) or DMSO (▴) as a control. Data show the mean ± SD of four experimental determinations.

The usefulness of any cellulose biosynthesis inhibitor to aid in the dissection of the biosynthetic pathway is governed by its ability to inhibit cellulose synthesis without inhibiting cell growth. To further validate pellicin as a useful tool for cellulose biosynthesis studies, its affect on the growth of *G. xylinus* was investigated (Fig. 3). The growth rate in the presence of pellicin was 1.5 times faster (μ = 0.040 h^−1^±0.01) compared to cells grown in the presence of dimethyl sulfoxide (DMSO) (μ = 0.026 h^−1^±0.01). The addition of DMSO did not alter the growth rate of *G. xylinus* under the growth conditions used in this study.

**Figure 4:**
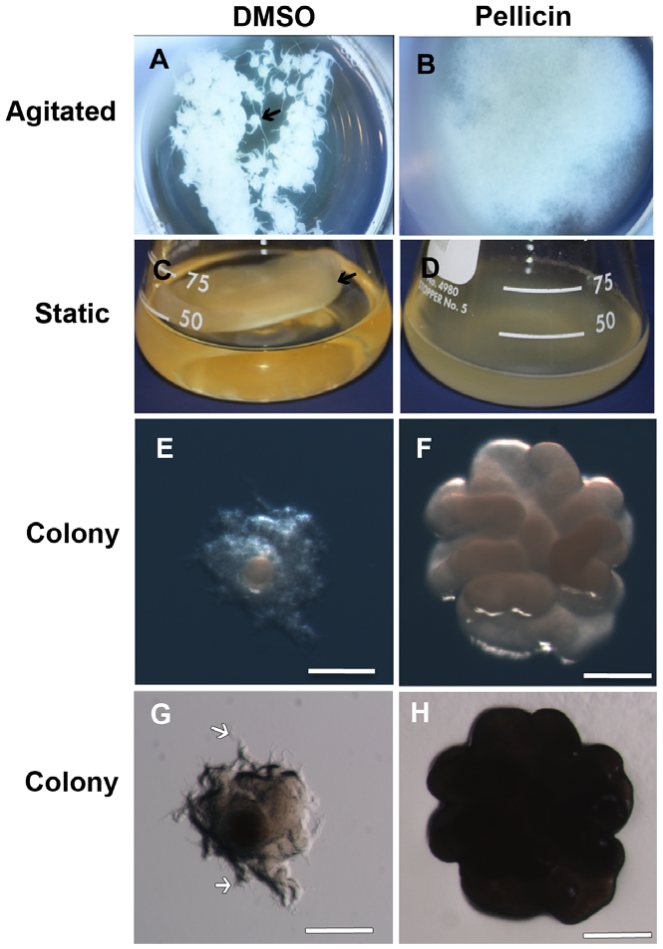
Effect of pellicin on cellulose production in liquid culture and solid medium. *G. xylinus was* grown at 30°C in liquid culture under agitated (A, B) and static conditions (C, D); (A, C) are DMSO controls, (B, D) grown in the presence of 10 µM pellicin. Arrows in (A, C) indicate either the large aggregates or pellicle that form in the absence of pellicin. Colony morphology of *G. xylinus* grown on SH agar plates that were supplemented with (E, G) DMSO or (F, H) 10 µM pellicin. Photographs of (E, F) were taken with illumination from above and (G, H) were taken with illumination from below of the same colonies. Note the larger, undulate, raised colonies forming on pellicin supplemented plates (F). Arrows in (G) indicate the filiform projections emerging from colony, which are absent in (H). Scale bars equal 0.5 mm.

**Figure 5:**
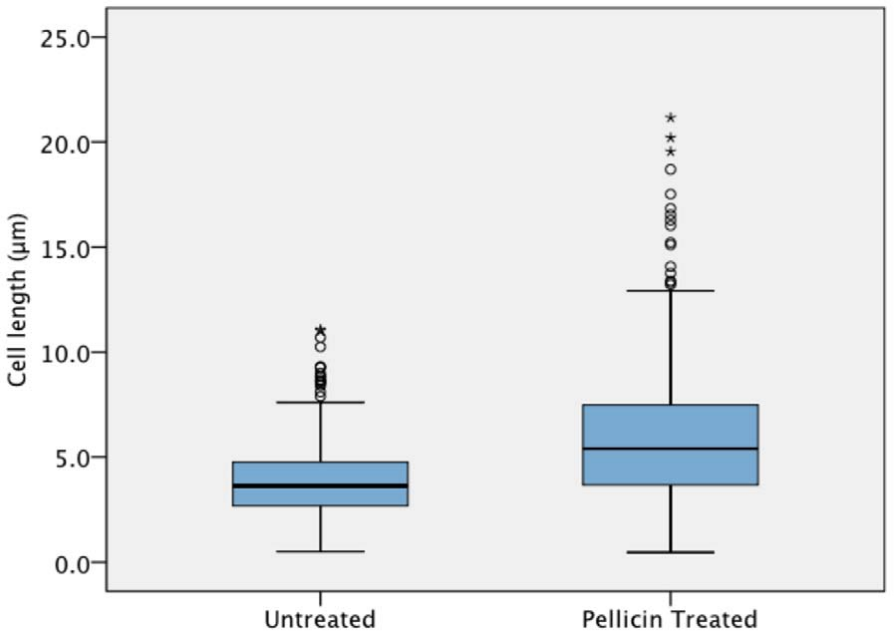
Pellicin affects the cell length of *G. xylinus*. Box plot of cell lengths of *G. xylinus* grown in the presence and absence of pellicin. The box signifies the upper and lower quartiles for each growth condition and the median is represented by the black line within each box. Outliers are denoted by (○). Extreme outliers are denoted by (*). Cell lengths of 500 cells from each growth condition were measured (p<0.001, independent samples *t*-test).

*G. xylinus* grown without inhibitor in agitated culture produced round balls of cellulose (Fig. 4A), while under static culture conditions a thick pellicle formed at the air-liquid interface (Fig. 4C). Within 72–96 h of agitated growth, the round cellulose balls would coalesce into one large aggregate of pellicle. No distinct pellicle was formed under static (Fig. 4B) or under agitated (Fig. 4D) culture conditions when pellicin was added to the culture medium. In the presence of pellicin, *G. xylinus* grew as dispersed cultures. Pellicin affected the colonial morphology of *G. xylinus* cultures. Colonies were notably larger, raised, undulate and smooth when grown in the presence of pellicin (Fig. 4E) compared to DMSO-grown controls which were rough and umbonate (Fig. 4F). In addition, while filiform projections were visible in DMSO controls (Fig. 4G) these were absent in pellicin-grown colonies (Fig. 4H). Microscopic examination of *G. xylinus* grown statically in liquid cultures treated with 0.1% cellulase to remove cellulose showed that pellicin treated cells were significantly longer than untreated cells and exhibited greater variability in cell length (Fig. 5; p<0.001, independent samples *t*-test).

### *In vitro* cellulose synthesis is unimpeded in the presence of pellicin

**Figure 6:**
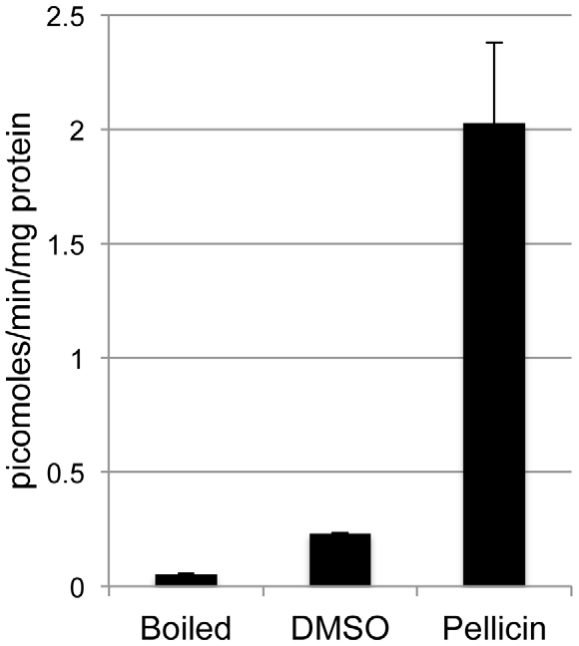
*In vitro* cellulose synthase assay using membrane preparations of *G. xylinus* grown in the presence and absence of pellicin using UDP-[^3^H]glucose as substrate. 1) Boiled control 2) Untreated 3) Pellicin pretreated. Cells grown in the presence of pellicin show an increased cellulose synthase activity. Data show the mean ± SE of three experimental determinations.

The use of cell-free preparations to measure cellulose synthesis *in vitro* has been well established [31], [32], [33]. To investigate the possible effect of pellicin on cellulose synthase activity, *in vitro* cellulose synthase assays using membrane preparations of *G. xylinus* grown in the presence and absence of pellicin with UDP-[^3^H]glucose as substrate were performed. The quantification of the radioactivity incorporated into the hot alkali-insoluble β-1,4-linked glucan product is a measure of cellulose II synthesis [34]. When *G. xylinus* cells were grown in the presence of pellicin and then used to prepare the crude membrane preparations an eight-fold increase in cellulose synthesis was observed compared to the amount observed for untreated membrane preparations (Fig. 6). Overall, these results showed that cellulose synthase activity was not directly inhibited by pellicin. This phenomenon was also apparent by the rapid production of cellulose observed when cells grown in the presence of pellicin were transferred to medium lacking pellicin. Pellicle production resumed at a rate that could not be accounted for by growth of the organism (data not shown). This observation provided indirect evidence that pellicin was not metabolized by *G. xylinus* cultures.

### Pellicin exerts its inhibitory effect outside the cell

**Figure 7:**
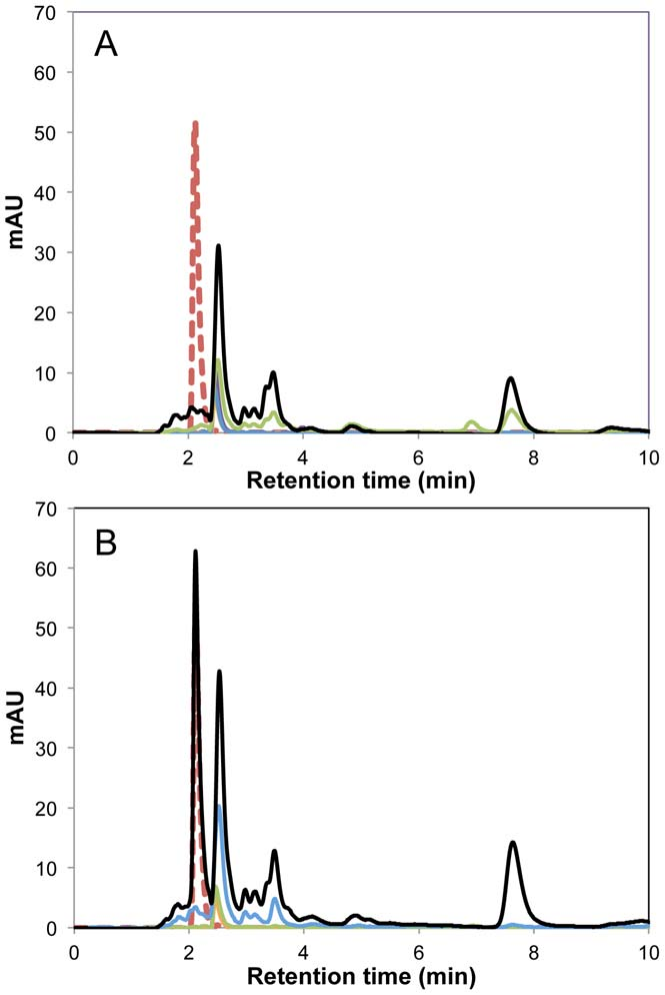
Pellicin remains in the extracellular environment. High performance liquid chromatography of organic extracts of A) *G. xylinus* in the absence and B) presence of pellicin. Data shown is representative of triplicate experiments: (red dashed line) pellicin standard, (black line) extracellular supernatant, (blue line) pellet wash prior to cellulase treatment, (green line) supernatant from cellulase treated cells, (orange line) cell free lysate, (purple line) cellular debris analyzed at 220 nm, the optimum wavelength for pellicin detection.

To further investigate the cellular location of pellicin activity, HPLC was used to analyse aqueous and organic extracts of intracellular and extracellular *G. xylinus* fractions. Pellicin was identified in the organic extracts of culture supernatant (Fig. 7) but was not detected in any other organic fraction confirming that it is not metabolized and therefore must exert its effect in the extracellular environment. Pellicin was not detected in any of the aqueous fractions (data not shown).

### Pellicin affects cellulose crystallinity

**Figure 8 pone-0028015-g008:**
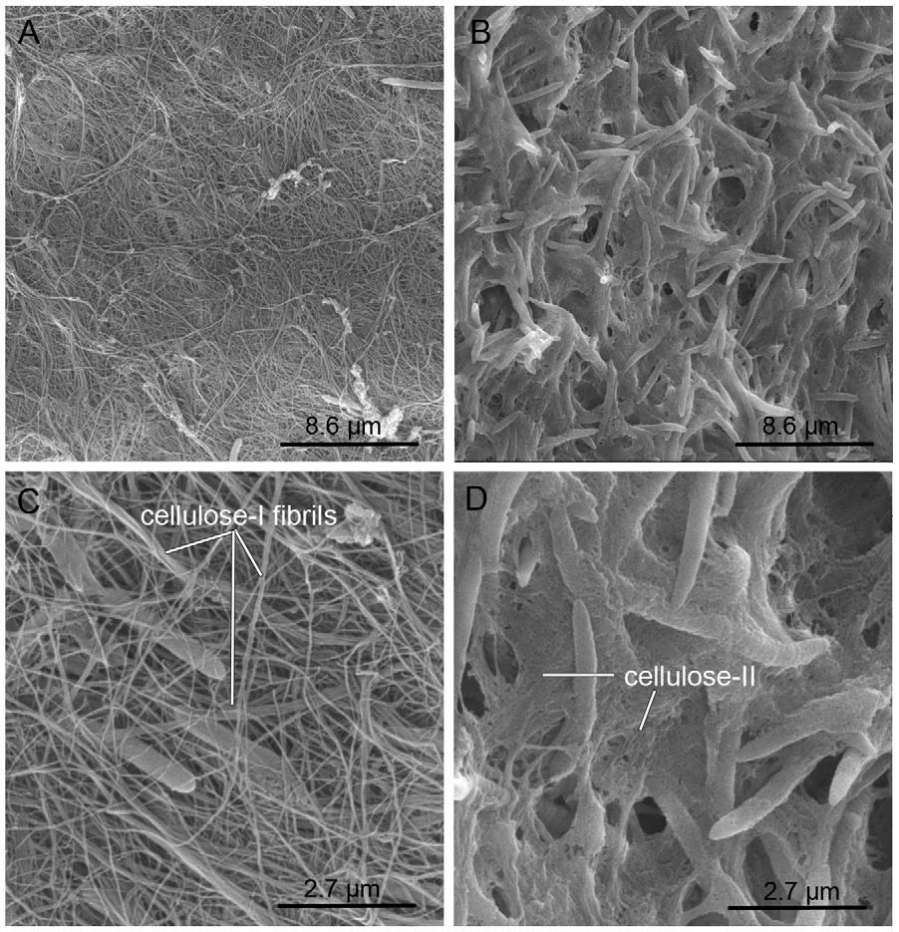
Pellicin affects the crystallinity of cellulose. Scanning electron micrographs of *G. xylinus* culture grown in the SH medium containing DMSO (A and C) and 10 µM pellicin (B and D). Untreated cultures have distinct, long cellulose-I fibrils that surround *G. xylinus* cells (C), while cultures treated with pellicin have shortened, cellulose-II strands that appears to be more gel-like (D). Scale bar are 8.6 µm in A and B and 2.7 µm in C and D.

In an effort to explain why the inhibitor, pellicin behaved like an activator of cellulose synthesis in the *in vitro* assays, scanning electron microscopy (SEM) was performed on pellicin treated and untreated cultures. SEM analysis revealed that an amorphous polysaccharide was produced in the presence of pellicin (Fig. 8) but that the long fibrils of cellulose characteristic of crystalline cellulose I production in *G. xylinus* were not formed (Fig. 8). These results suggest that cellulose II biosynthesis was proceeding normally but that final assembly into the highly crystalline form, cellulose I, was affected by pellicin. The observed increase in cellulose synthase activity may be an attempt by cells to compensate for the lack of crystallinity. This might also explain the rapid production of pellicle upon transfer to medium devoid of inhibitor.

**Figure 9:**
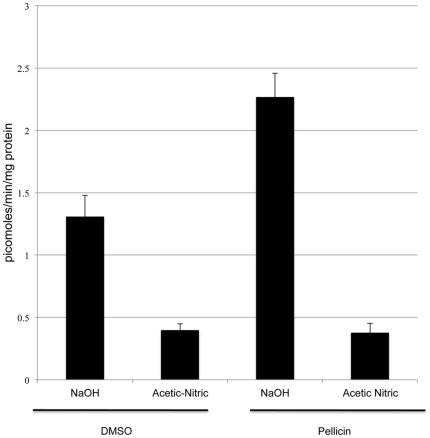
Pellicin affects the crystallization of cellulose produced by *G. xylinus*. *In vitro* cellulose synthase assays of crude enzyme prepared from *G. xylinus* grown SH medium and incubated with DMSO or pellicin for 1 hour prior to UDP-[^3^H]glucose addition. Reactions treated with 0.5 M NaOH contain both cellulose I and cellulose II. Acetic-nitric acid treatment of reactions removes cellulose II while retaining cellulose I. Data show the mean ± SE of three experimental determinations.

To further explore the impact of pellicin on cellulose crystallinity, the *in vitro* assays were repeated, this time incorporating a modification to include acetic and nitric acid to remove cellulose II, as described by Updegraff [35]. In addition, we assayed whether addition of pellicin to the reaction mixture would have an effect on cellulose production. In this manner, we determined that indeed, pellicin increases the amount of cellulose II formation over non-pellicin treated cells, while the more crystalline form, cellulose I was produced in equivalent amounts regardless of the presence of inhibitor (Fig. 9).

**Figure 10:**
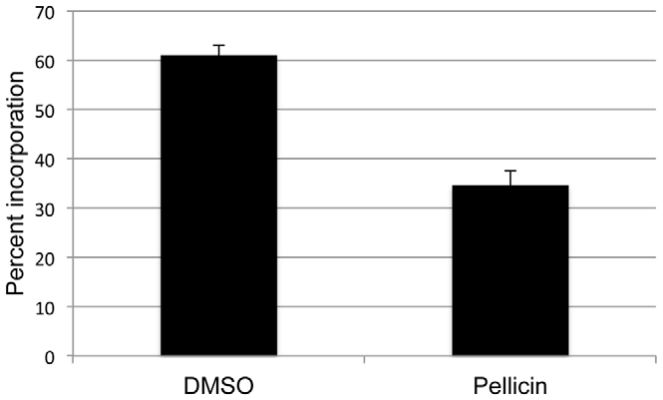
Relative proportion of cellulose I to cellulose II produced during *in vitro* cellulose synthesis assay. The ratio of cellulose I to cellulose II production was determined from crude enzyme preparations of *G. xylinus* grown in DMSO and 10 µM pellicin by assaying the amount of UDP-[^3^H]glucose in the acid insoluble and soluble fractions as described in the Materials and Methods.

Quantitation of radioactive incorporation into the acid insoluble fraction and acid soluble fraction of membrane preparations from pellicin untreated and treated cells provided a measure of the relative amounts of cellulose synthesis *in vitro*. It was determined that cellulose synthase from untreated cells synthesized 1.6 times more crystalline cellulose than cellulose synthase from pellicin treated cells (Fig. 10).

**Figure 11:**
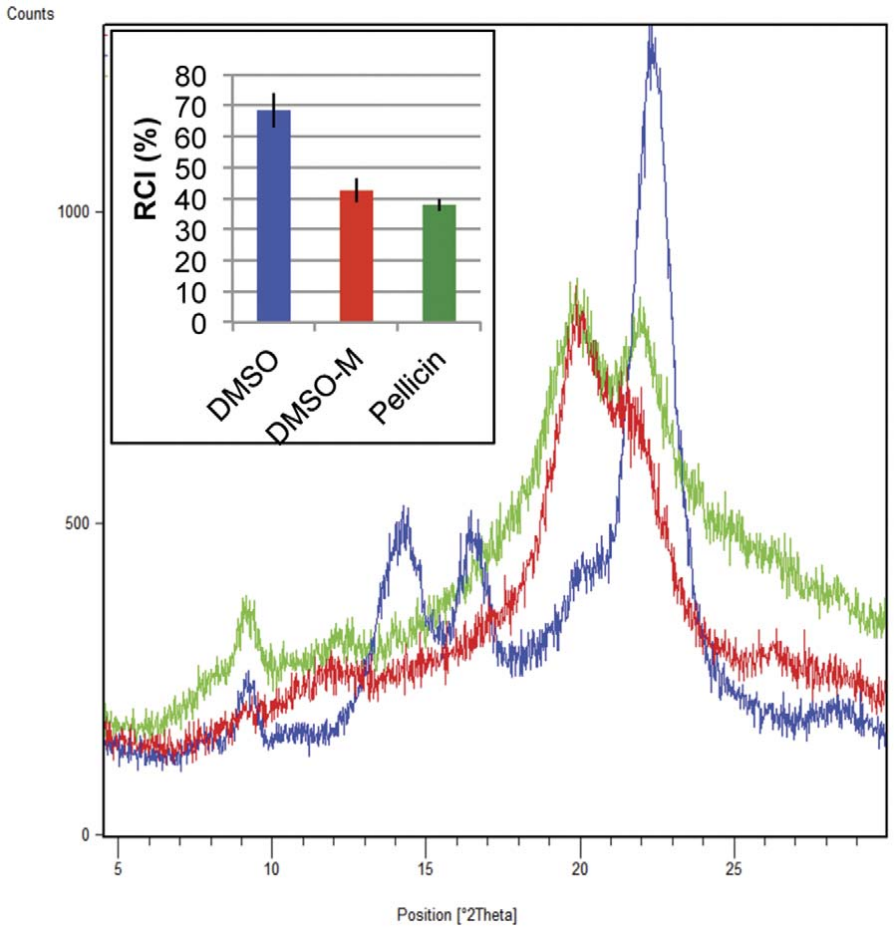
Powder X-ray diffraction pattern for extracellular cellulose produced by *G. xylinus* grown in the absence (blue) or presence of 10 µM pellicin (green) compared to mercerized bacterial cellulose (red). Inset, relative crystallinity index (RCI) of cellulose produced under different conditions calculated from X-ray diffraction (see Materials and Methods for details). Values ± SD are shown; n = 3 for each treatment.

Further confirmation that pellicin affects cellulose crystallinity but not cellulose synthase activity was provided by powder X-ray diffraction (XRD) [36]. XRD analysis showed that pellicle produced in the absence of pellicin was highly crystalline as evidenced by the intense peak at 2θ of 22° compared with less crystalline cellulose produced by cells grown in the presence of pellicin. Mercerization converts cellulose I to cellulose II [5]. When pellicle from untreated *G. xylinus* was mercerized, the X-ray diffraction pattern closely resembled the pattern observed for pellicin treated cells, notably, the loss of the intense peak at 2θ of 22° (Fig. 11). The relative crystallinity index (RCI) of *G. xylinus* pellicle was close to 70% compared to the RCI values of mercerized pellicle and pellicin grown cells, which were 42% and 37% respectively.

## Discussion

Although structurally cellulose is a simple homopolysaccharide, many details regarding its biosynthesis and extracellular assembly have remained elusive. The identification of pellicin as an inhibitor of pellicle formation provides a useful tool for investigating the extracellular assembly of cellulose II into the crystalline, cellulose I form.

The rate of cellulose production in *G. xylinus* has previously been reported to be proportional to the rate of cell growth [37]. Pellicin affected the cellular morphology of *G. xylinus* resulting in longer cells and a faster growth rate. Although we can only speculate at this point, the observed filamentation phenotype uncovered by pellicin might suggest a potential connection between cellular division and cellulose biosynthesis in *G. xylinus*. A connection between cell division and cellulose biosynthesis has been reported for *Escherichia coli* [38], [39] and *Streptomyces* [40]. It is also likely, however, that the cell elongation is the result of some secondary effect of pellicin that we are not yet aware of.

We have established that cellulose synthase activity was not directly affected by pellicin treatment. There was no pellicin dose-dependent [^3^H]UDP-glucose incorporation into cellulose observed *in vitro* (Fig. 6). Rather, pellicin was found to affect the crystallinity of cellulose produced by *G. xylinus* after the pre-cellulosic ß-1,4-glucan chains, called tactoidal aggregates, were formed and extruded extracellularly (Figs. 7–9).

It is known that cellulose polymerization occurs at the membrane [32], [41]. While the biochemistry of the polymerization reaction is poorly understood, polymerization and crystallization are thought to be tightly coupled events [42], [43]. Tactoidal aggregates assemble into crystalline microfibrils while their composite ß-1,4-glucan chains are still undergoing elongation [43]. In the present study, we found that the final microfiber formation is inhibited by pellicin (Fig. 8 and Fig. 9). Interestingly, in the presence of pellicin, the rate of cellulose synthesis increases just as is observed for Calcofluor White ST, a fluorescent brightener which also disrupts the final microfiber formation [43]. Unlike Calcofluor White ST [44], cell growth is not inhibited by pellicin suggesting greater specificity for cellulose.

Calcofluor White ST forms hydrogen bonds with β-1,4-glucans [45], [46] in a dose-dependent manner [44], [45]. If pellicin disrupted hydrogen bonding, one would expect that as cellulose synthesis continues in an active culture, more binding sites would become available to bind the pellicin. Eventually, the free pellicin concentration would decrease in the medium with a concomitant increase of normal hydrogen bonding and therefore some normal crystalline pellicle would form. This is not, however, observed. Pellicin inhibition of crystallization remains stable even in active cultures over 6 months old. Furthermore, pellicin is only detectable in the culture supernatant. The conclusion from this is that pellicin does not have the same mechanism of action as Calcofluor White ST for the disruption of cellulose crystallization.

Given that pellicin addition to the *in vitro* cellulose synthase assays had a minor stimulatory effect (Fig. 9), we conclude that pellicin binds an extracellular membrane component, likely a protein. Just as the disruption of crystallinity by Calcofluor White ST is reversible, so too is the action of pellicin. Pellicin decouples polymerization from crystallization. Crystallization slows down the rate of cellulose production and influences the rate of polymerization [43]; this is also consistent with our results (Fig. 6 and Fig. 9). Participation of proteins yet to be implicated in the process must be involved in the organization of the final cellulose ribbon on the extracellular surface of the cell, likely as part of a protein complex at the extrusion site of the pre-cellulosic polymers. For example, BcsD and CCPax have been proposed to influence crystallization of cellulose [15] but neither has been shown to directly participate in the process. Identification of the target for pellicin would provide insight into the mechanism by which the tactoidal aggregates are assembled into the crystalline microfibrils.

## Materials and Methods

### Bacteria, medium and growth conditions

*Gluconacetobacter xylinus* ATCC 53582 was cultivated in Schramm-Hestrin (SH) medium [47] either statically or on a rotary shaker at 28°C. To obtain a uniform suspension of *G. xylinus* cells devoid of cellulose, 0.1% (v/v) cellulase (Sigma, St. Louis, Mo) was added to growth medium to digest the cellulose. To obtain growth curve data, 96-well microtitre plates were inoculated with a total volume of 150 µL SH medium containing 0.1% cellulase and either DMSO or 10 µM pellicin dissolved in DMSO. The starting inoculum level was adjusted to an optical density of 0.05 for both culture conditions. Plates were incubated at 28°C with shaking at 150 rpm. Optical density was recorded at 600 nm using a Bio-Rad xMark™ Microplate Absorbance Spectrophotometer (Bio-Rad Laboratories Ltd., Mississauga, ON).

### Chemical Genetic Screen for identification of cellulose biosynthesis inhibitors

To identify the bacterial cellulose inhibitor, 10,000 individual DIVERSet library compounds (30–50 µM) were screened (plates 10626–10750). To do this, a 96-well formatted library was diluted into liquid media and inoculated with *G. xylinus* cells using a replicator comb. Bacterial cells were incubated at 28°C for five days until a cellulose pellicle had formed in the majority of wells. Compounds that prevented pellicle formation but not cell growth were retested. Those compounds that inhibited cellulose synthesis were validated by measuring cellulose according to Updegraff [35]. As controls, *G. xylinus* cells were grown in liquid media containing 0.1% cellulase (Sigma, St. Louis, MO). Cell density was determined by measuring the absorbance at 600 nm (OD_600_).

### Scanning electron microscopy

Washed pellicle or cells were fixed in FAA fixative (50% ethanol, 5% acetic acid, 3.7% formalin) overnight. Fixed pellicle/cells were dehydrated through a graded series of water-ethanol solutions with the ethanol increasing 10% (v/v) each step. At the end, dehydrated sample was washed twice in absolute ethanol. Samples were then immersed in hexamethyldisilazane for 5 min followed by air drying for 10 min, which were then coated in gold. Observations were made using a Hitachi S-570 scanning electron microscope.

### Fluorescence microscopy

Cellular morphology of *G. xylinus* grown in the presence and absence of 10 µM pellicin was observed using a Nikon Optihot fluorescent microscope at a magnification of 1000×. Both cultures were treated with 0.1% cellulase to digest cellulose. A 1∶1 volumetric ratio of aqueous propidium iodide (Sigma Chemical Co., St. Louis, Mo.) at a concentration of 5 mg/mL was added to cell suspensions. Cell lengths were determined for 500 cells of each culture type in three biological replicates using Macnification software (Orbicule, Inc., Belgium). Cell length data were analyzed using an independent sample t-test to compare the length of cells grown in the presence and absence of pellicin. The graphical and statistical analyses were performed using PASW Statistics 18.0 (IBM Corporation, Somers, NY, USA).

### Preparation and analysis of *G. xylinus* extracts

*G. xylinus* was grown in SH medium containing DMSO or 10 µM pellicin in triplicate cultures. Pellicle and cells respectively were separated from culture supernatant, washed and digested with cellulase. Cells were passed through a French pressure cell (Thermo Electron) at 1280 psi and centrifuged at 12,000×g for 10 min to remove cell debris. Culture supernatant, cell washes, cell debris and cell free extract were freeze dried, resuspended in 30% acetonitrile and extracted with an equal volume of methylene chloride. Uninoculated medium containing DMSO or 10 µM pellicin were also extracted in a similar manner. Organic and aqueous extracts were evaporated to dryness, resuspended in 30% acetonitrile, filtered through 0.2 µm filters and analyzed using a Shimadzu Prominence HPLC system equipped with a PDA detector using a 5 µm apHeraTM C18 column (Sepulco) 150×4.6 mm at a flow rate of 0.8 mL/min with isocratic elution in 30% acetonitrile. A pellicin standard was used to determine that the optimum wavelength for detection was 220 nm.

### Particulate-membrane enzyme preparation

*Gluconacetobacter xylinus* was grown in SH medium containing DMSO or 10 µM pellicin dissolved in DMSO for 72 h. Pellicle was digested by the addition of 0.1% (v/v) cellulase. Cells were harvested by centrifugation and washed with cold Tris-hydrochloride buffer (pH 7.5) containing 10 mM MgCl_2_ and 1 mM EDTA (TME buffer) as previously described [32]. All steps for preparation of the crude particulate-membrane separation were performed at 4°C unless otherwise specified. Washed cells were resuspended in TME buffer containing 20% (w/v) PEG-4000 passed through a French pressure cell (Thermo Electron) at 1280 psi and centrifuged at 12,000×*g* for 10 min. The pellet containing the membrane fraction was suspended in TME buffer to one-fourth the original volume using a Teflon homogenizer and centrifuged at 1,500×*g* for 3 min. The protein content of the supernatant containing the membrane fraction was determined by the method of Bradford [48] using bovine serum albumin as the standard protein after adjusting the PEG-4000 to a final concentration of 8% (v/v).

### *In vitro* cellulose synthase assays

Cellulose synthase assays were carried out at 30°C in a total reaction volume of 0.2 mL as described by Aloni et al. [32]. Each reaction contained the following: 80 µL TME buffer, 20 µL of 0.5 M Tris-HCl (pH 9.6) containing 50 mM MgCl_2_ and 5 mM EDTA, 0.5 µM UDP-[^3^H]glucose, 0.25 mM GTP and 100 µg of crude enzyme. After incubation for 15 min, the reaction was terminated by the addition of 1.5 mL of a solution containing 0.5 M NaOH, 0.5 M NaBH_4_ and 20 mg carrier cellulose at 100°C for 20 min. In some cases,1 µM pellicin was added to the reaction mixture along with all other components and incubated for 1 h prior to the addition of UDP-[^3^H]glucose. The extracts were then filtered through Whatman GF/A glass fibre filters and washed six times with 4 mL of H_2_O followed by a final wash with 4 mL methanol. The filters were dried at 60°C and the radioactivity present in the alkali-insoluble product (total cellulose) was determined by scintillation counting in a Perkin Elmer Tri-Carb 2800 TR Liquid Scintillation Analyzer. Crystalline cellulose was determined by treating the reaction mixture with hot 58% acetic acid and 19% nitric acid [35], [49]. The acid insoluble material representing the crystalline cellulose and the soluble material were separated by filtration through glass fibre filters as described above. The amount of label present in each fraction was determined by scintillation counting.

### X-ray Diffraction

X-ray diffraction was used to assess the crystallinity of cellulose as previously described [36]. Samples were prepared by lyophilizing pellicle of *G. xylinus* grown in SH medium containing DMSO or cell pellet of cells grown in the presence of 10 µM pellicin. Lyophilized material was finely ground and examined by Powder X-ray diffraction (XRD) using a Philips XRD system (Department of Geology, University of Toronto, Canada). Powdered microcrystalline cellulose (Avicel, Sigma, St. Louis, Mo.) and carboxymethylcellulose (Bioshop, Burlington, Ontario) were used as reference samples. The relative crystallinity index [36], RCI was calculated using the formula RCI = (I_002_−I_am_/I_002_)×100, where I_002_ is the overall intensity of the peak at 2θ of 22° and I_am_ is the intensity of the baseline at 2θ of 18°.
